# Supplementary material for: RING finger protein 13 protects against nonalcoholic steatohepatitis by targeting STING-relayed signaling pathways
Source: Nat Commun. 2023 Oct 20;14:6635. doi: 10.1038/s41467-023-42420-1 (PMC10587083; doi:10.1038/s41467-023-42420-1)
Supplement: Supplementary file 1 — Supplementary Information [file 41467_2023_42420_MOESM1_ESM.pdf]

Supplementary Materials for

RING finger protein 13 protects against nonalcoholic steatohepatitis by targeting STING-relayed signaling pathways

Zhi-Bin Lin *et al.*

Correspondence: fierywang@163.com

This PDF file includes:

Supplementary Figure 1-8

Supplementary Table 1-5

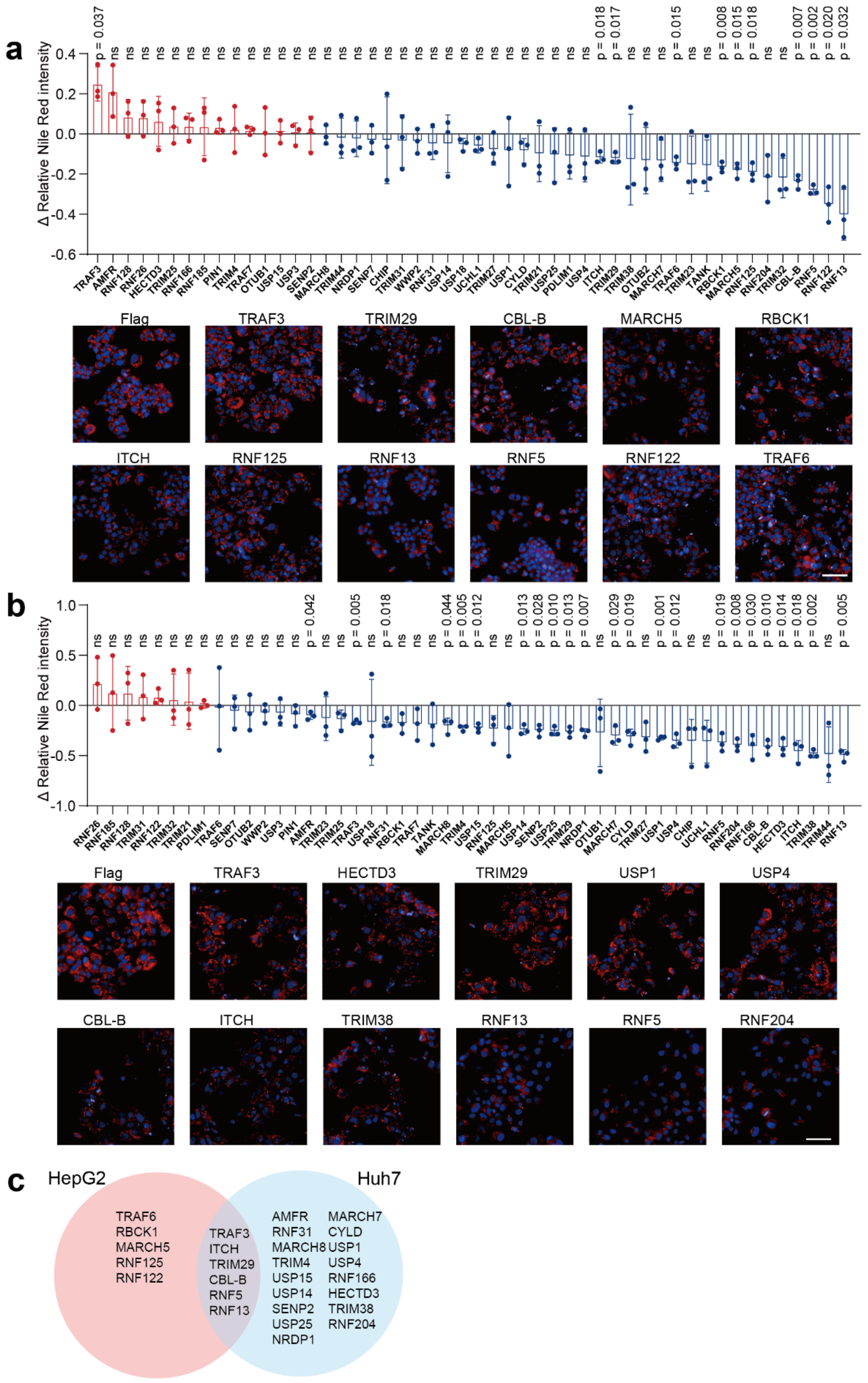
a
Δ Relative Nile Red intensity
TRAF3 AMFR RNF128 RNF26 HECTD3 TRIM25 RNF166 RNF185 PIN1 TRIM4 TRAF7 OTUB1 USP15 USP3 SENP2 MARCH8 TRIM44 NRDP1 SENP7 CHIP TRIM31 WWP2 RNF31 USP14 USP18 UCHL1 TRIM27 USP1 CYLD TRIM21 USP25 PDLIM1 USP4 ITCH TRIM29 TRIM38 OTUB2 MARCH7 TRAF6 TRIM23 TANK RBCK1 MARCH5 RNF125 RNF204 TRIM32 CBL-B RNF5 RNF122 RNF13
Flag
TRAF3
TRIM29
CBL-B
MARCH5
RBCK1
ITCH
RNF125
RNF13
RNF5
RNF122
TRAF6
b
Δ Relative Nile Red intensity
RNF26 RNF185 RNF128 TRIM31 RNF122 TRIM32 TRIM21 PDLIM1 TRAF6 SENP7 OTUB2 WWP2 USP3 PIN1 AMFR TRIM23 TRIM25 TRAF3 USP18 RNF31 RBCK1 TRAF7 TANK MARCH8 TRIM4 USP15 RNF125 MARCH5 USP14 SENP2 USP25 TRIM29 NRDP1 OTUB1 MARCH7 CYLD TRIM27 USP1 USP4 CHIP UCHL1 RNF5 RNF204 RNF166 CBL-B HECTD3 ITCH TRIM38 TRIM44 RNF13
Flag
TRAF3
HECTD3
TRIM29
USP1
USP4
CBL-B
ITCH
TRIM38
RNF13
RNF5
RNF204
c
HepG2
Huh7
TRAF6
RBCK1
MARCH5
RNF125
RNF122
TRAF3
ITCH
TRIM29
CBL-B
RNF5
RNF13
AMFR
RNF31
MARCH8
TRIM4
USP15
USP14
SENP2
USP25
NRDP1
MARCH7
CYLD
USP1
USP4
RNF166
HECTD3
TRIM38
RNF204

Supplementary Fig. 1. Phenotype-based high-content screening of innate immunity-related E3 ligase and deubiquitinating enzymes. High-content screening based on Nile Red staining was performed by using HepG2 cell line (**a**) and Huh7 cell line (**b**) (n=3). Cells were transfected with the indicated plasmids for 24 hours and then treated with PAOA for 12 hours. The upper panels show the alteration of relative Nile Red intensity by plasmids transfecting, and the lower panels show the representative images. Nuclei were counterstained with DAPI. Scale bars, 50 μm. **c** A Venn diagram showing proteins significantly affect the lipid disposition in PAOA-challenged HepG2 and Huh7 cell lines. Data were expressed as mean ± SD. Two-tailed Student's t test for **a** and **b**. Source data are provided as a Source Data file.

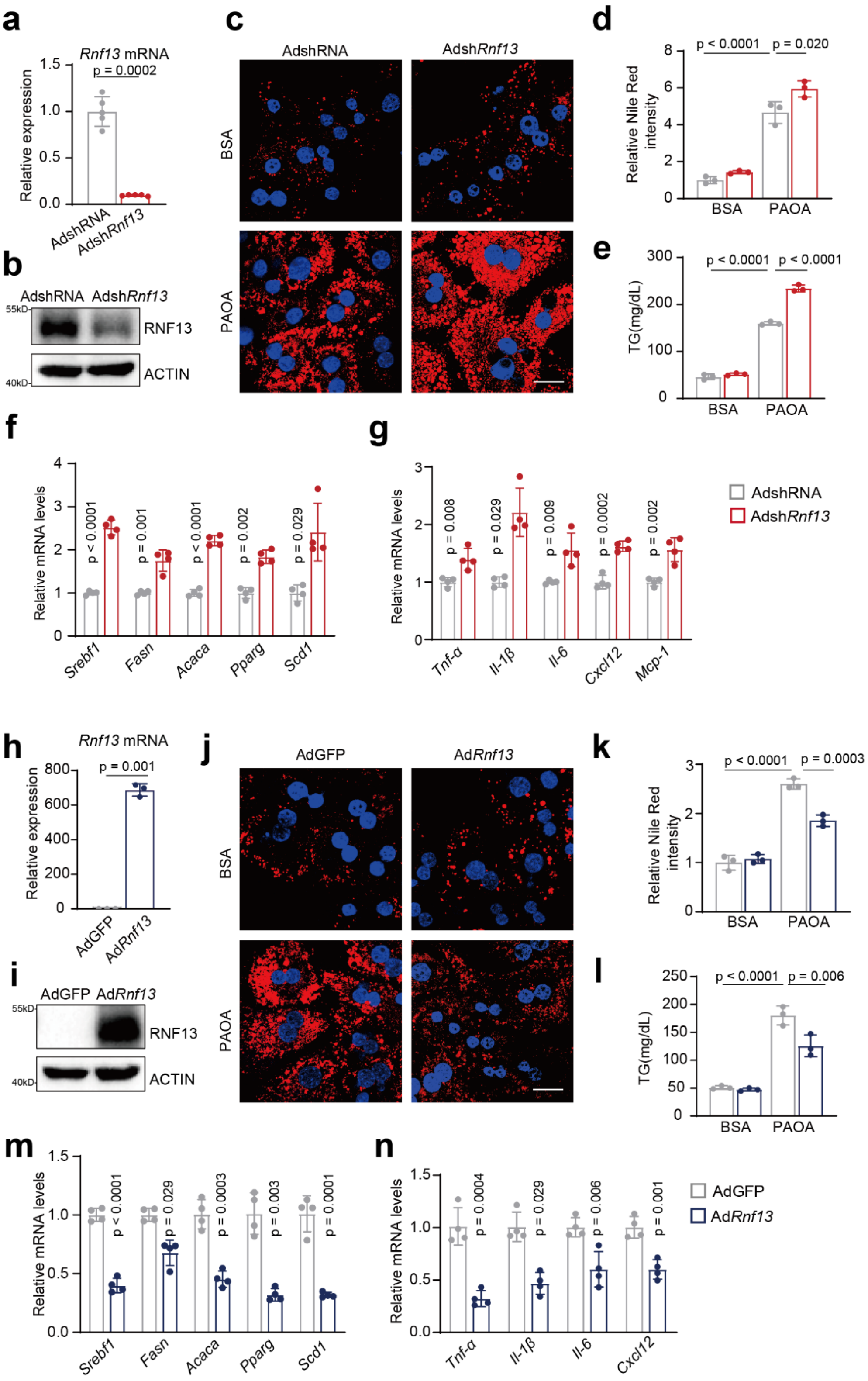
a
Rnf13 mRNA
p = 0.0002
Relative expression
AdshRNA
AdshRnf13
b
AdshRNA AdshRnf13
55kD
RNF13
ACTIN
40kD
c
AdshRNA
AdshRnf13
BSA
PAOA
d
p < 0.0001 p = 0.020
Relative Nile Red intensity
BSA PAOA
e
p < 0.0001 p < 0.0001
TG(mg/dL)
BSA PAOA
f
Relative mRNA levels
p < 0.0001
p = 0.001
p < 0.0001
p = 0.002
p = 0.029
Srebf1
Fasn
Acaca
Pparg
Scd1
g
Relative mRNA levels
p = 0.008
p = 0.029
p = 0.009
p = 0.0002
p = 0.002
Tnf-α
Il-1β
Il-6
Cxcl12
Mcp-1
AdshRNA
AdshRnf13
h
Rnf13 mRNA
p = 0.001
Relative expression
AdGFP
AdRnf13
i
AdGFP AdRnf13
55kD
RNF13
ACTIN
40kD
j
AdGFP
AdRnf13
BSA
PAOA
k
p < 0.0001 p = 0.0003
Relative Nile Red intensity
BSA PAOA
l
p < 0.0001 p = 0.006
TG(mg/dL)
BSA PAOA
m
Relative mRNA levels
p < 0.0001
p = 0.029
p = 0.0003
p = 0.003
p = 0.0001
Srebf1
Fasn
Acaca
Pparg
Scd1
n
Relative mRNA levels
p = 0.0004
p = 0.029
p = 0.006
p = 0.001
Tnf-α
Il-1β
Il-6
Cxcl12
AdGFP
AdRnf13

Supplementary Fig. 2. RNF13 inhibits PAOA-induced lipogenesis and inflammation in hepatocytes. *Rnf13* mRNA (**a**) and protein levels (**b**) in MPHs infected with AdshRNA or Adsh*Rnf13* (n=5). Nile Red staining (**c**), quantification (**d**) and TG contents (**e**) of MPHs infected with AdshRNA or Adsh*Rnf13* following BSA or PAOA treatment for 12 hours (n=3). Nuclei were counterstained with DAPI. Scale bars, 25 μm. qPCR analyses of lipogenic (**f**) and proinflammatory (**g**) gene expression in MPHs infected with AdshRNA or Adsh*Rnf13* following 12-hour PAOA treatment (n=4). *Rnf13* mRNA (**h**) and protein levels (**i**) in MPHs infected with AdGFP or Ad*Rnf13* (n=3). Nile Red staining (**j**), quantification (**k**) and TG contents (**l**) of MPHs infected with AdGFP or Ad*Rnf13* following BSA or PAOA treatment for 12 hours (n=3). Nuclei were counterstained with DAPI. Scale bars, 25 μm. qPCR analyses of lipogenic (**m**) and proinflammatory (**n**) gene expression in MPHs infected with AdGFP or Ad*Rnf13* following 12-hour PAOA treatment (n=4). Data were expressed as mean ± SD. Two-tailed Student's t test for **a**, **f**, **g**, **h**, **m** and **n**, one-way ANOVA with Bonferroni post hoc analysis for **d**, **e**, **k** and **l**. Source data are provided as a Source Data file.

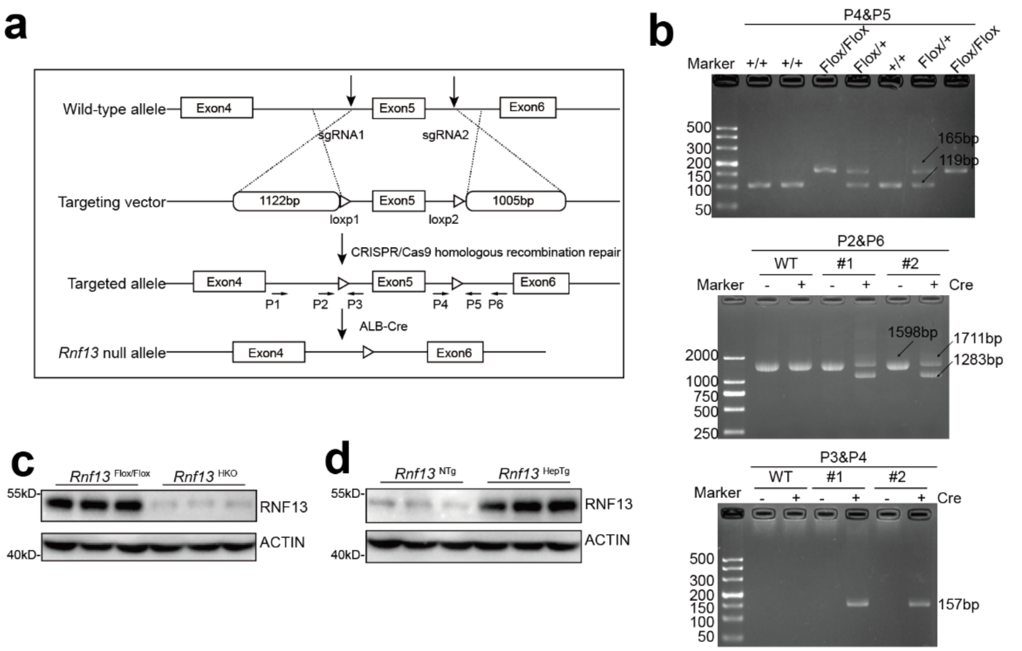

Supplementary Fig. 3. Generation of *Rnf13*$^{\mathbf{Flox/Flox}}$, *Rnf13*$^{\mathbf{HKO}}$, *Rnf13*$^{\mathbf{NTg}}$ and *Rnf13*$^{\mathbf{HepTg}}$ mice. a Schematic depiction of the generation of *Rnf13* hepatocyte-specific knockout mice (*Rnf13*^{HKO}) and the control mice (*Rnf13*$^{\mathrm{Flox/Flox}}$). **b** Genotyping of the *Rnf13*^{HKO} and *Rnf13*$^{\mathrm{Flox/Flox}}$ mice. **c** RNF13 protein level in the liver of the *Rnf13*^{HKO} and *Rnf13*$^{\mathrm{Flox/Flox}}$ mice (n=3). **d** RNF13 protein level in the liver of the *Rnf13*^{NTg} and *Rnf13*^{HepTg} mice (n=3). Source data are provided as a Source Data file.

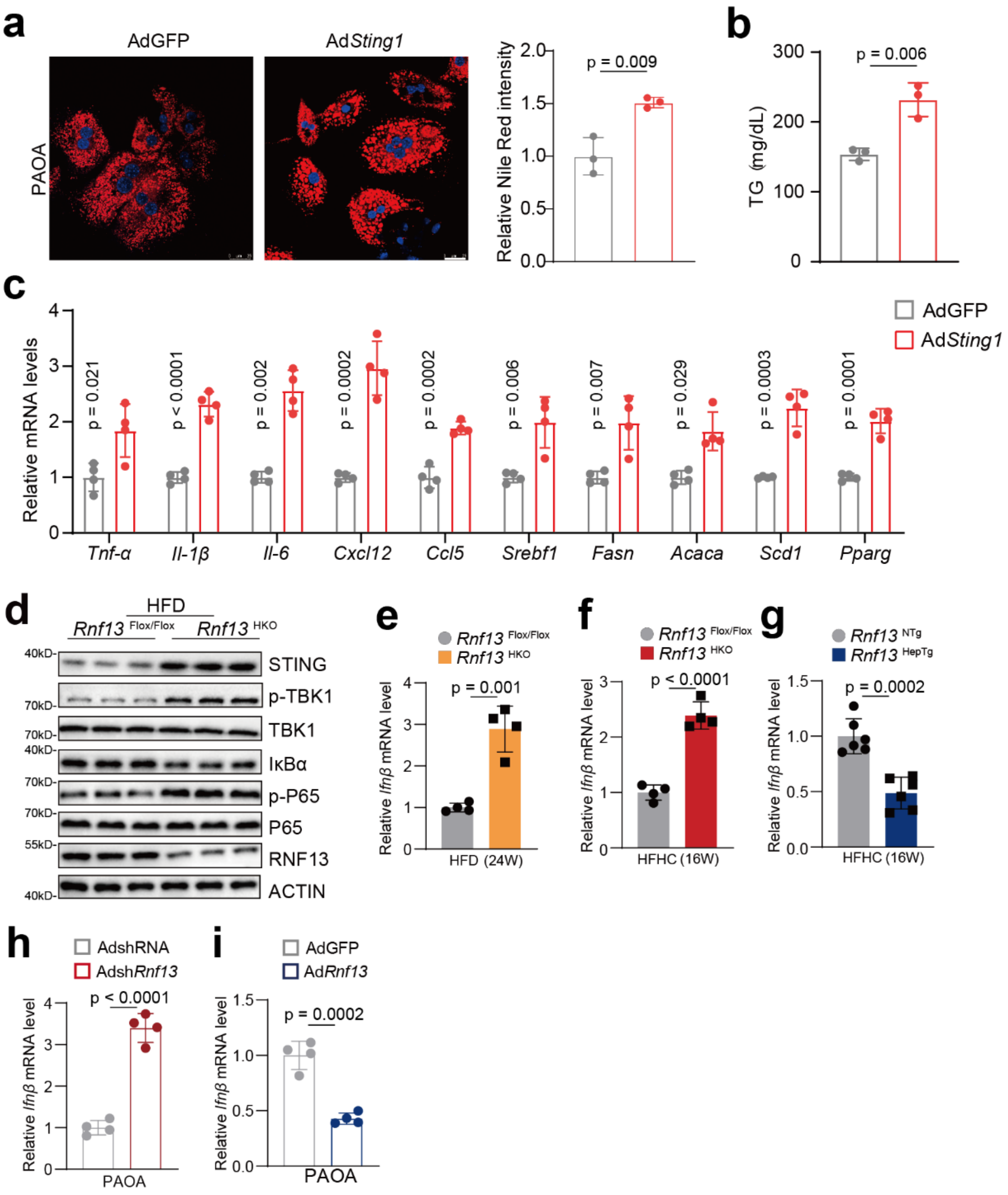
a
AdGFP
AdSting1
PAOA
Relative Nile Red intensity
p = 0.009
b
TG (mg/dL)
p = 0.006
c
Relative mRNA levels
AdGFP
AdSting1
p = 0.021
p < 0.0001
p = 0.002
p = 0.0002
p = 0.0002
p = 0.006
p = 0.007
p = 0.029
p = 0.0003
p = 0.0001
Tnf-α
Il-1β
Il-6
Cxcl12
Ccl5
Srebf1
Fasn
Acaca
Scd1
Pparg
d
HFD
Rnf13 Flox/Flox
Rnf13 HKO
STING
p-TBK1
TBK1
IκBα
p-P65
P65
RNF13
ACTIN
e
Rnf13 Flox/Flox
Rnf13 HKO
Relative Ifnβ mRNA level
p = 0.001
HFD (24W)
f
Rnf13 Flox/Flox
Rnf13 HKO
p < 0.0001
HFHC (16W)
g
Rnf13 NTg
Rnf13 HepTg
p = 0.0002
HFHC (16W)
h
AdshRNA
AdshRnf13
p < 0.0001
PAOA
i
AdGFP
AdRnf13
p = 0.0002
PAOA

Supplementary Fig. 4. Overexpressing STING in hepatocytes exacerbates PAOA-induced lipid accumulation and inflammation. Nile Red staining as well as quantification (**a**) and TG contents (**b**) of MPHs infected with AdGFP or Ad*Sting1* following PAOA treatment for 12 hours (in the presence of nonparenchymal cells) (n=3). Nuclei were counterstained with DAPI. Scale bars, 25 μm. **c** qPCR analyses of proinflammatory and lipogenic gene expression in MPHs infected with AdGFP or Ad*Sting1* following 12-hour PAOA treatment (in the presence of nonparenchymal cells) (n=4). **d** the protein level of RNF13, STING, p-P65/P65, IκBα and p-TBK1/TBK1 in the livers of the *Rnf13*$^{\mathrm{Flox/Flox}}$ and *Rnf13*^{HKO} mice fed with HFD for 24 weeks (n=3). The mRNA level of *Ifnβ* in the livers of the *Rnf13*$^{\mathrm{Flox/Flox}}$ and *Rnf13*^{HKO} mice fed with HFD for 24 weeks (**e**) or HFHC for 16 weeks (**f**) (n=4). **g** the mRNA level of *Ifnβ* in the livers of *Rnf13*^{NTg} and *Rnf13*^{HepTg} mice fed with HFHC for 16 weeks (n=6). The mRNA level of *Ifnβ* in MPHs infected with Adsh*Rnf13* (**h**) or Ad*Rnf13* (**i**) as well as the control adenovirus following PAOA treatment for 12 hours (n=4). Data were expressed as mean ± SD. Two-tailed Student's t test for all comparation. Source data are provided as a Source Data file.

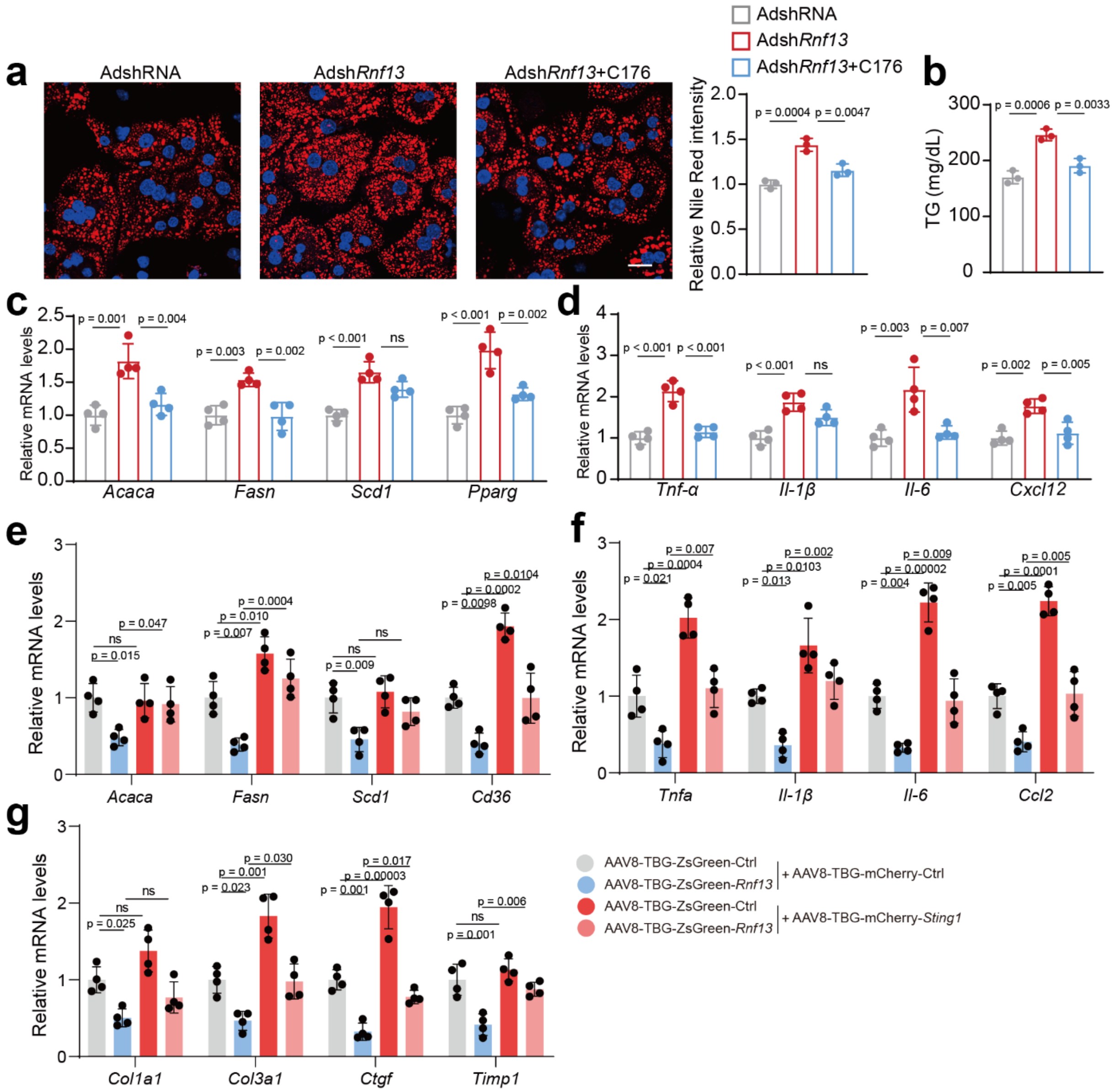
a
AdshRNA
Adsh*Rnf13*
Adsh*Rnf13*+C176
AdshRNA
Adsh*Rnf13*
Adsh*Rnf13*+C176
Relative Nile Red intensity
p = 0.0004
p = 0.0047
b
TG (mg/dL)
p = 0.0006
p = 0.0033
c
Relative mRNA levels
p = 0.001
p = 0.004
p = 0.003
p = 0.002
p < 0.001
ns
p < 0.001
p = 0.002
Acaca
Fasn
Scd1
Pparg
d
Relative mRNA levels
p < 0.001
p < 0.001
p < 0.001
ns
p = 0.003
p = 0.007
p = 0.002
p = 0.005
Tnf-α
Il-1β
Il-6
Cxcl12
e
Relative mRNA levels
p = 0.047
ns
p = 0.015
p = 0.0004
p = 0.010
p = 0.007
ns
ns
p = 0.009
p = 0.0104
p = 0.0002
p = 0.0098
Acaca
Fasn
Scd1
Cd36
f
Relative mRNA levels
p = 0.007
p = 0.0004
p = 0.021
p = 0.002
p = 0.0103
p = 0.013
p = 0.009
p = 0.00002
p = 0.004
p = 0.005
p = 0.0001
p = 0.005
Tnfa
Il-1β
Il-6
Ccl2
g
Relative mRNA levels
ns
ns
p = 0.025
p = 0.030
p = 0.001
p = 0.023
p = 0.017
p = 0.00003
p = 0.001
p = 0.006
ns
p = 0.001
Col1a1
Col3a1
Ctgf
Timp1
AAV8-TBG-ZsGreen-Ctrl
AAV8-TBG-ZsGreen-*Rnf13*
+ AAV8-TBG-mCherry-Ctrl
AAV8-TBG-ZsGreen-Ctrl
AAV8-TBG-ZsGreen-*Rnf13*
+ AAV8-TBG-mCherry-*Sting1*

Supplementary Fig. 5. RNF13 ameliorates lipogeneses and inflammation through STING. Nile Red staining as well as quantification (**a**), TG contents (**b**), lipogenic (**c**) and proinflammatory gene expression (**d**) of MPHs infected with AdshRNA or Adsh*Rnf13* following PAOA treatment for 8 hours (in the presence of nonparenchymal cells). In the meantime, MPHs were treated with DMSO or C176 (1 μM). Nuclei were counterstained with DAPI (For **a** and **b**, n=3; for **c** and **d**, n=4). Scale bars, 25 μm. Lipogenetic (**e**), proinflammatory (**f**) and profibrotic (**g**) mRNA expression in the liver of mice from the indicated groups (n=4). Data were expressed as mean ± SD. One-way ANOVA with Bonferroni post hoc analysis for all comparation. Source data are provided as a Source Data file.

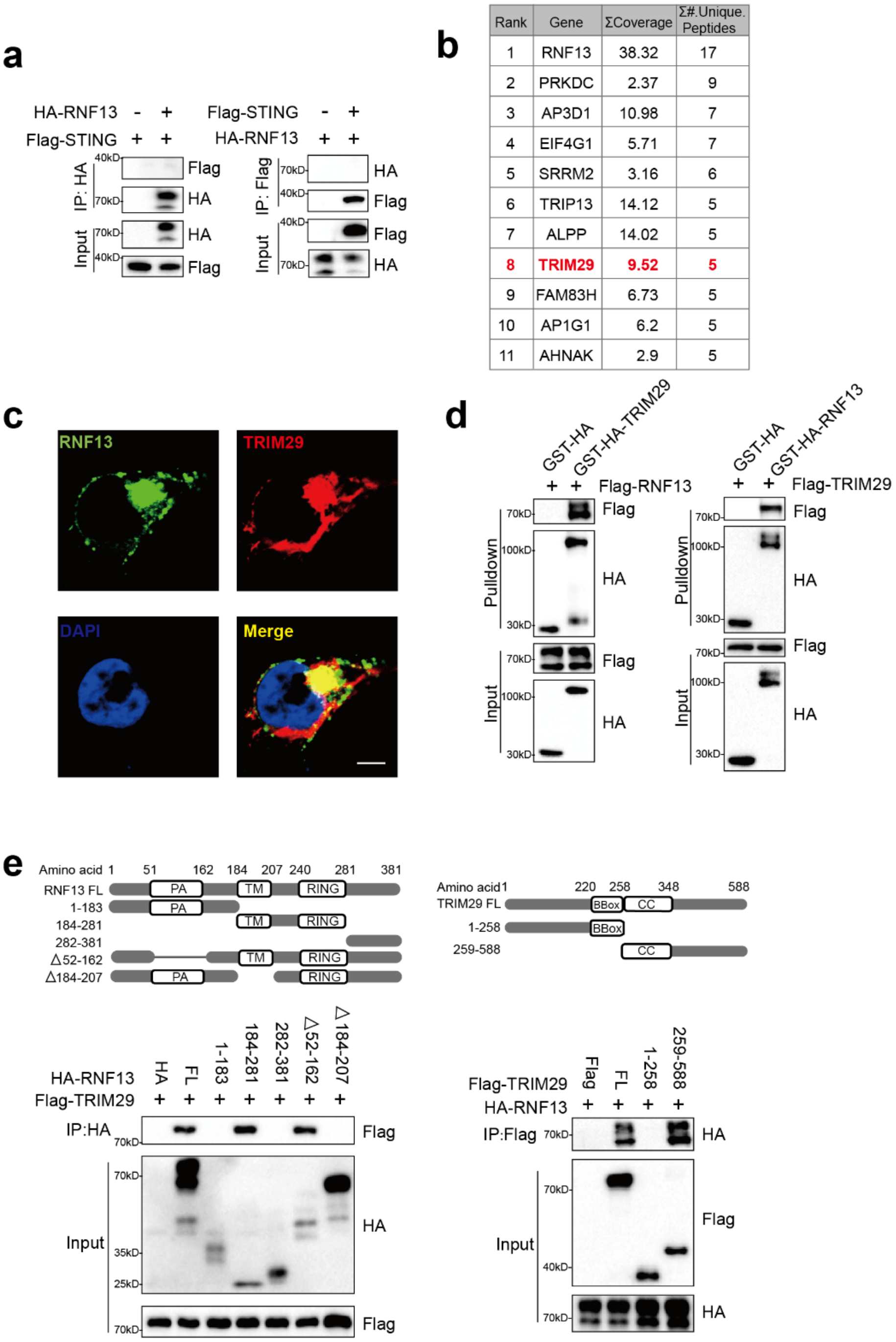

Rank	Gene	ΣCoverage	Σ#.Unique. Peptides
1	RNF13	38.32	17
2	PRKDC	2.37	9
3	AP3D1	10.98	7
4	EIF4G1	5.71	7
5	SRRM2	3.16	6
6	TRIP13	14.12	5
7	ALPP	14.02	5
8	TRIM29	9.52	5
9	FAM83H	6.73	5
10	AP1G1	6.2	5
11	AHNAK	2.9	5

Supplementary Fig. 6. RNF13 interacts with TRIM29. a Co-immunoprecipitation of Flag-STING and HA-RNF13 in HEK293T cells co-transfected with the indicated plasmids. **b** The result of IP-MS. Proteins are ranked by the number of unique peptides. **c** Immunofluorescent staining of HA-RNF13 (green) and Flag-TRIM29 (red) in HEK293T cells co-transfected with the indicated plasmids. Nuclei were counterstained with DAPI. Scale bars, 5 μm. In vitro pull-down assays (**d**) and molecular mapping assays (**e**) of RNF13 and TRIM29 in HEK293T cells co-transfected with the indicated plasmids. For **a** and **c-e**, at least three independent experiments have been conducted. Source data are provided as a Source Data file.

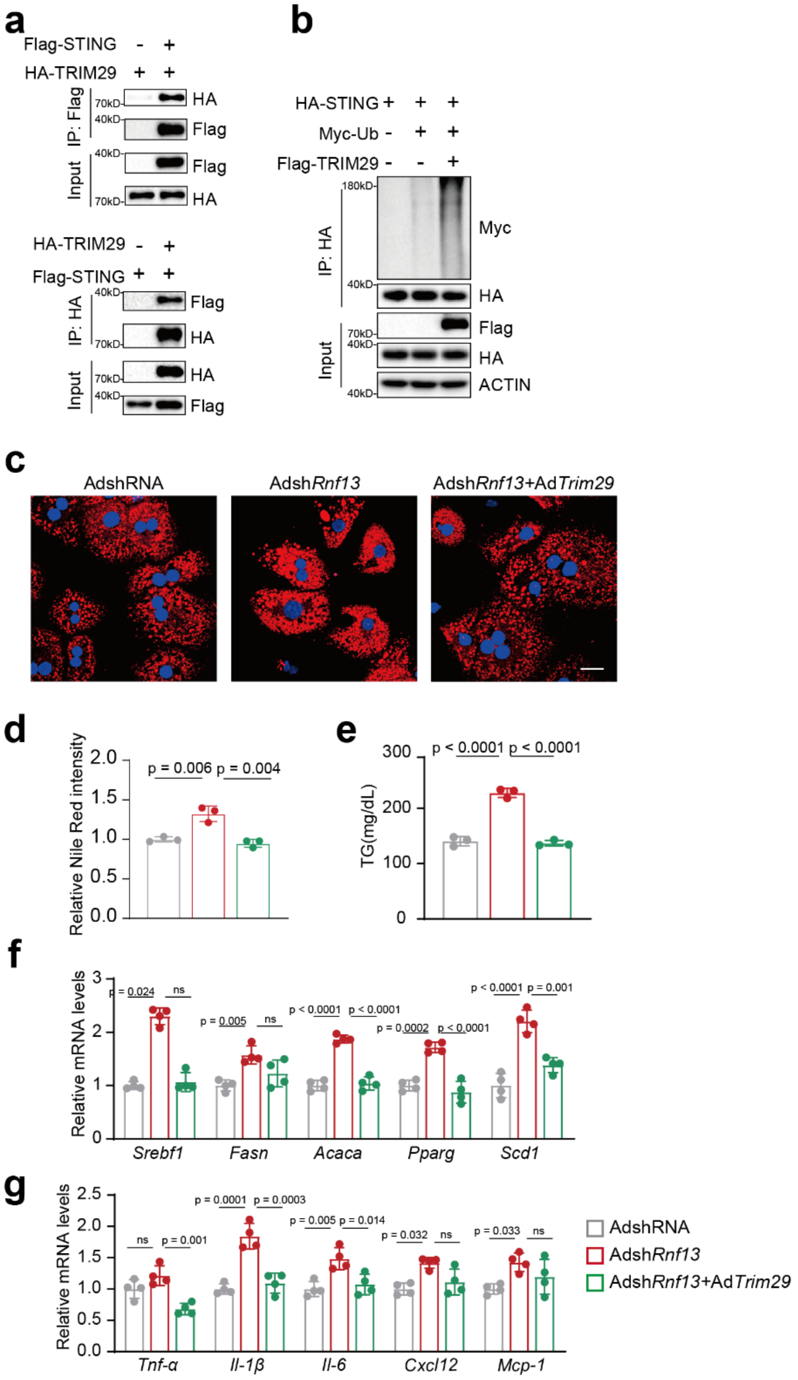
a
Flag-STING - +
HA-TRIM29 + +
IP: Flag
Input
HA
Flag
Flag
HA
HA-TRIM29 - +
Flag-STING + +
IP: HA
Input
Flag
HA
HA
Flag
b
HA-STING + + +
Myc-Ub - + +
Flag-TRIM29 - - +
IP: HA
Input
Myc
HA
Flag
HA
ACTIN
c
AdshRNA
AdshRnf13
AdshRnf13+AdTrim29
d
Relative Nile Red intensity
p = 0.006
p = 0.004
e
TG(mg/dL)
p < 0.0001
p < 0.0001
f
Relative mRNA levels
Srebf1
Fasn
Acaca
Pparg
Scd1
g
Relative mRNA levels
Tnf-α
Il-1β
Il-6
Cxcl12
Mcp-1
AdshRNA
AdshRnf13
AdshRnf13+AdTrim29

Supplementary Fig. 7. TRIM29 is the downstream effector of RNF13 for STING degradation. a Co-immunoprecipitation of Flag-STING and HA-TRIM29 in HepG2 cells co-transfected with the indicated plasmids. **b** Ubiquitination of exogenous STING in HepG2 cells co-transfected with the indicated plasmids, followed by PAOA plus MG132 treatment. For **a** and **b**, at least three independent experiments have been conducted. Nile Red staining (**c**) and quantification (**d**), TG contents (**e**), lipogenic (**f**) and proinflammatory gene expression (**g**) in MPHs infected with AdshRNA, Adsh*Rnf13* or Adsh*Rnf13* plus Ad*Trim29*, followed by PAOA treatment. Scale bars, 25μm. For **c-e**, n=3; for **f-g**, n=4. Data were expressed as mean ± SD. One-way ANOVA with Bonferroni post hoc analysis for all comparation. Source data are provided as a Source Data file.

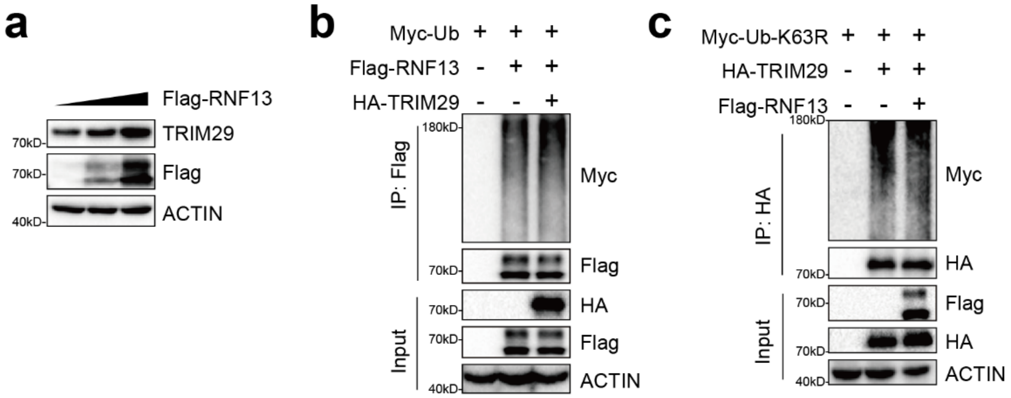

Supplementary Fig. 8. RNF13 stabilizes TRIM29 by enhancing the K63-linked ubiquitination of TRIM29. a Endogenous TRIM29 protein expression in HepG2 cells in response to different dose of RNF13 overexpression. **b** Ubiquitination of Flag-RNF13 in HepG2 cells co-transfected with the indicated plasmids, followed by PAOA plus MG132 treatment. **c** Ubiquitination of HA-TRIM29 in HepG2 cells co-transfected with the indicated plasmids, followed by PAOA plus MG132 treatment. For **a-c**, at least three independent experiments have been conducted. Source data are provided as a Source Data file.

Supplementary Table 1. Clinical characteristics of individuals included in the Figure 1b-d.

Index/Group	NASH	Non-NASH	P value
Gender	Male (6); Female (9)	Male (6); Female (11)	N/A
Age	36.00(24.00, 52.00)	46.00(36.00, 58.00)	P=0.261
BMI	29.97(27.58, 36.82)	25.22(23.36, 28.70)	P=0.005
NAS	5.00(4.00, 6.00)	1.00(0.00, 1.00)	P<0.001
ALT	35.00(19.00,77.00)	18.00(9.50, 50.00)	P=0.142
AST	33.00(18.00, 62.00)	19.50(14.25, 30.50)	P=0.165
TBIL	15.00(10.90, 18.50)	12.00(9.17, 17.28)	P=0.142
ALP	86.00(61.00, 135.00)	73.50(53.50, 79.50)	P=0.040
γ-GT	46.00(19.00, 92.00)	32.00(13.50, 51.25)	P=0.049

Data are shown as median (first quartile, third quartile).

Supplementary Table 2. sgRNA target sequences and primers for transgenic animal construction.

sgRNA target sequences for knockout mice construction

sgRNA	Target Sequence (5’-3’)
sgRNA1	GATGCCTATAGGAGAACACCTAGG
sgRNA2	GATAAAAGACAAGTCAATTCAACCTTGG

Primers for mice identification

**Primer**	**Primer Sequence (5’-3’)**
**P2**	GAAATGAGAAAGCATCTAAAGACC
**P3**	TGCCTAGAAGCACACCATGT
**P4**	GGTCTTGGGGTACTCTTAATTCC
**P5**	TTTGAAAGAAAATATCCACAGATAAA
**P6**	CTGTGCTGCATCGTACCCTA

Supplementary Table 3. Primers for plasmid construction.

Primer sequences for overexpressing plasmids

Gene	Forward (5'-3')	Reverse (5'-3')
RNF13-HA	TCGGGTTTAAACGGATCCGCCACCATGCTGCTCTCCATAGGGATGC	GGGCCCTCTAGACTCGAGAACAGTATTTGCTATGTTGTA
RNF13-Flag	TCGGGTTTAAACGGATCCGCCACCATGCTGCTCTCCATAGGGATGC	GGGCCCTCTAGACTCGAGAACAGTATTTGCTATGTTGTA
RNF13(C258A/H260A)	GACAAACTCAGAATCCTTCCCGCATCCGCTGCTTATCATTGCAAG	CTTGCAATGATAAGCAGCGGATGCGGGAAGGATTCTGAGTTTGTC
RNF13-GST-HA	TCGGGTTTAAACGGATCCGCCACCATGCTGCTCTCCATAGGGATGC	GGGCCCTCTAGACTCGAGAACAGTATTTGCTATGTTGTA
RNF13(1-183)	TCGGGTTTAAACGGATCCGCCACCATGCTGCTCTCCATAGGGATGC	GGGCCCTCTAGACTCGAGGTAGTATTCCAAAGGAAGACTAAATTCTGG
RNF13(184-281)	TCGGGTTTAAACGGATCCGCCACCATGCTAATTCCCTTCCTTATCATAGTGGGCA	GGGCCCTCTAGACTCGAGGCACACTGGACAGGTTTTTTTGG

RNF13(282-381)	TCGGGTTTAAACGGATCCGCCACCATGAAGCAAAAAGTTGTTCCTTCTCAAGGC	GGGCCCTCTAGACTCGAGAACAGTATTTGCTATGTTGTA
RNF13(Δ52-162)	TCAGACATTTGATGACCTCACATATGAAAAAGGGGGCCACC	TTTCATATGTGAGGTCATCAAATGTCTGAGATGCATT
RNF13(Δ184-207)	GGAATACTACCAGGATAGACATAGAGCTAGAAGAAACAG	GTCTATCCTGGTAGTATTCCAAAGGAAGACTAAATTCTG
STING1-HA	TCGGGTTTAAACGGATCCATGCCCCACTCCAGCCTG	GGGCCCTCTAGACTCGAGTCAAGAGAAATCCGTGCGGAG
STING1-Flag	TCGGGTTTAAACGGATCCATGCCCCACTCCAGCCTG	GGGCCCTCTAGACTCGAGTCAAGAGAAATCCGTGCGGAG
TRIM29-HA	TCGGGTTTAAACGGATCCATGGAAGCTGCAGATGCCTC	GGGCCCTCTAGACTCGAGTCATGGGGCTTCGTTGGACC
TRIM29-Flag	TCGGGTTTAAACGGATCCATGGAAGCTGCAGATGCCTC	GGGCCCTCTAGACTCGAGTCATGGGGCTTCGTTGGACC

TRIM29-GST-HA	TCGGGTTTAAACGGATCCATGGAAGCTGCAGATGCCTC	GGGCCCTCTAGACTCGAGTCATGGGGCTTCGTTGGACC
TRIM29(1-258)	TCGGGTTTAAACGGATCCATGGAAGCTGCAGATGCCTC	GGGCCCTCTAGACTCGAGTCACACGGTGCTATGATTCTTGTGC
TRIM29(259-588)	TCGGGTTTAAACGGATCCATGACAGTGGAGGAGGCCAAG	GGGCCCTCTAGACTCGAGTCATGGGGCTTCGTTGGACC

Primer sequences for adenoviral plasmids

Gene	Forward (5'-3')	Reverse (5'-3')
Rnf13	GGCTAGCGATATCGGATCCATGCTGCTCTCCATTGGGAT	CGTCCTTGTAATCACTAGTCACAGTATTTGCTATGTTGTAATCC
Rnf13(C258A/H260A)	GATCCTCCCCGCATCCGCTGCTTATCATTGCAAGTGTGTAGATCCCTG	ATGATAAGCAGCGGATGCGGGGAGGATCCTTAGCTTATCTCCG
Sting1	GGCTAGCGATATCGGATCCGCCACCATGCCATACTCCAACCTGCA	CGTCCTTGTAATCACTAGTGATGAGGTCCGTGCGGAG
Trim29	GGCTAGCGATATCGGATCCGCCACCATGGAAGGTGCCGATGCCT	CGTCCTTGTAATCACTAGTGGGCGCCTCGTTGGATCC

Primer sequences for plasmids used in phenotype-based screening

Gene	Forward (5’-3’)	Reverse (5’-3’)
TRIM4	TCGGGTTTAAACGGATCCATGGACGGCAGTGGACCCTT	GGGCCCTCTAGACTCGAGTCACCTCCTCTTCAGCACGG
RNF31	TCGGGTTTAAACGGATCCATGCCGGGGGAGGAAGAGGA	GGGCCCTCTAGACTCGAGCTACTTCCGCCTGCGGGGGA
AMFR	TCGGGTTTAAACGGATCCATGCCGCTGCTCTTCCTCGA	GGGCCCTCTAGACTCGAGCTAGGAGGTCTGCTGCTTCT
CBLB	TCGGGTTTAAACGGATCCATGGGCTATTTGTGTGTTAATTTCA	GGGCCCTCTAGACTCGAGCTATAGATTTAGACGTGGGGATACTG
RNF5	TCGGGTTTAAACGGATCCATGGCAGCAGCGGAGGAGGA	GGGCCCTCTAGACTCGAGTCAAATACTGAGCAGCCAAA
TRIM21	TCGGGTTTAAACGGATCCATGGCTTCAGCAGCACGCTTGACAA	GGGCCCTCTAGACTCGAGTCAATAGTCAGTGGATCCTTGTG
HECTD3	TCGGGTTTAAACGGATCCATGGCGGGTCCTGGCCCGGG	GGGCCCTCTAGACTCGAGTCACTCCTCCCAAGGGCTCATG
CHIP	TCGGGTTTAAACGGATCCATGAAGGGCAAGGAGGAGAAGG	GGGCCCTCTAGACTCGAGTCAGTAGTCCTCCACCCAGCCA
TRAF7	TCGGGTTTAAACGGATCCATGAGCTCAGGCAAGAGTGC	GGGCCCTCTAGACTCGAGTTAGCAAGTCCAAACCTTCACAG
USP4	TCGGGTTTAAACGGATCCATGGCGGAAGGTGGAGGCT	GGGCCCTCTAGACTCGAGTTAGTTGGTGTCCATGCTGCAAGC
USP25	TCGGGTTTAAACGGATCCATGACCGTGGAGCAGAATGTG	GGGCCCTCTAGACTCGAGTTATCTTCCATCAGCAGGCG

RNF125	TCGGGTTTAAACGGATCCATGGG CTCCGTGCTGAGCAC	GGGCCCTCTAGACTCGAGTTATG TGGTGTTCGAGTGAT
CYLD	TCGGGTTTAAACGGATCCATGAG TTCAGGCTTATGGAG	GGGCCCTCTAGACTCGAGTTATT TGTACAAACTCATTGTT
RNF13	ACGCGTGGTCTCGGATCCAAAAA AGCAGGCACCATG	TCTAGACGTCTCCTCGAGAACAG TATTTGCTATGTTGTAATC
USP3	ACGCGTGGTCTCGGATCCAAAAA AGCAGGCACCATG	TCTAGACGTCTCCTCGAGAAGTT TATCCGATCCAGCTTTG
TANK	ACGCGTGGTCTCGGATCCAAAAA AGCAGGCACCATG	TCTAGACGTCTCCTCGAGAATGC TGCTTTCTGCAGAAGC
RNF128	ACGCGTGGTCTCGGATCCAAAAA AGCAGGCACCATG	TCTAGACGTCTCCTCGAGAGATT TAATTTCTCGAACAGCAG
TRIM23	ACGCGTGGTCTCGGATCCAAAAA AGCAGGCACCATG	TCTAGACGTCTCCTCGAGAGCAA CATCCAATACTCCAGC
TRIM27	ACGCGTGGTCTCGGATCCAAAAA AGCAGGCACCATG	TCTAGACGTCTCCTCGAGAGGGG AGGTCTCCATGGAAT
RNF122	ACGCGTGGTCTCGGATCCAAAAA AGCAGGCACCATG	TCTAGACGTCTCCTCGAGCACCA GCTCATCCAATAGAAT
SENP2	ACGCGTGGTCTCGGATCCAAAAA AGCAGGCACCATG	TCTAGACGTCTCCTCGAGCAGCA ACTGCTGATGAAGGA
WWP2	ACGCGTGGTCTCGGATCCAAAAA AGCAGGCACCATG	TCTAGACGTCTCCTCGAGCCCTG GAGGAAGGGGCC

PIN1	ACGCGTGGTCTCGGATCCAAAAA AGCAGGCACCATG	TCTAGACGTCTCCTCGAGCTCAG TGCGGAGGATGATGT
ITCH	ACGCGTGGTCTCGGATCCAAAAA AGCAGGCACCATG	TCTAGACGTCTCCTCGAGCTCTT GTCCAAATCCTTCTGT
RNF204	ACGCGTGGTCTCGGATCCAAAAA AGCAGGCACCATG	TCTAGACGTCTCCTCGAGCTGAC CAGGGGCCTCAGG
USP14	ACGCGTGGTCTCGGATCCAAAAA AGCAGGCACCATG	TCTAGACGTCTCCTCGAGCTGTT CACTTTCCTCTTCCAT
OTUB2	ACGCGTGGTCTCGGATCCAAAAA AGCAGGCACCATG	TCTAGACGTCTCCTCGAGCTTGA AGATCTCCCTGCTCT
PDLIM1	ACGCGTGGTCTCGGATCCAAAAA AGCAGGCACCATG	TCTAGACGTCTCCTCGAGCTTGG GGAACACAGTGACCA
TRIM25	ACGCGTGGTCTCGGATCCAAAAA AGCAGGCACCATG	TCTAGACGTCTCCTCGAGCTTGG GGGAGCAGATGGAG
MARCH8	ACGCGTGGTCTCGGATCCAAAAA AGCAGGCACCATG	TCTAGACGTCTCCTCGAGGACGT GAATGATTTCTGCTCC
RNF26	ACGCGTGGTCTCGGATCCAAAAA AGCAGGCACCATG	TCTAGACGTCTCCTCGAGGAGGT AGACATTGAGGGTCT
USP18	ACGCGTGGTCTCGGATCCAAAAA AGCAGGCACCATG	TCTAGACGTCTCCTCGAGGCACT CCATCTTCATGTAAAC
SENP7	ACGCGTGGTCTCGGATCCATGGA CAAGAGAAAGCTCGGG	TCTAGACGTCTCCTCGAGGCTAC TGCTGCCCTTCTGTTG

TRIM31	ACGCGTGGTCTCGGATCCAAAAAAGCAGGCACCATG	TCTAGACGTCTCCTCGAGGCTTGAAGGAACCTCACAAAA
MARCH7	ACGCGTGGTCTCGGATCCATGGAGTCTAAACCTTCAAGGATTCCAA	TCTAGACGTCTCCTCGAGGGCAATATCAAATGTCCTGTTATGGTCT
RNF185	ACGCGTGGTCTCGGATCCAAAAAAGCAGGCACCATG	TCTAGACGTCTCCTCGAGGGCAATCAGGAGCCAGAACA
UCHL1	ACGCGTGGTCTCGGATCCAAAAAAGCAGGCACCATG	TCTAGACGTCTCCTCGAGGGCTGCCTTGCAGAGAGC
TRAF3	ACGCGTGGTCTCGGATCCAAAAAAGCAGGCACCATG	TCTAGACGTCTCCTCGAGGGGATCGGGCAGATCCGA
TRIM38	ACGCGTGGTCTCGGATCCAAAAAAGCAGGCACCATG	TCTAGACGTCTCCTCGAGGTCACCTGGGGGAGGCAG
RBCK1	ACGCGTGGTCTCGGATCCAAAAAAGCAGGCACCATG	TCTAGACGTCTCCTCGAGGTGGCAGTTCTGACAGCTTG
USP15	ACGCGTGGTCTCGGATCCAAAAAAGCAGGCACCATG	TCTAGACGTCTCCTCGAGGTTAGTGTGCATACAGTTTTCA
RNF166	ACGCGTGGTCTCGGATCCAAAAAAGCAGGCACCATG	TCTAGACGTCTCCTCGAGGTTCTCAGAGAGAGACAGGG
USP1	ACGCGTGGTCTCGGATCCAAAAAAGCAGGCACCATG	TCTAGACGTCTCCTCGAGTAATTTCTTATAAAATAGCAAGTAAG
TRAF6	ACGCGTGGTCTCGGATCCAAAAAAGCAGGCACCATG	TCTAGACGTCTCCTCGAGTACCCCTGCATCAGTACTTC

NRDP1	ACGCGTGGTCTCGGATCCAAAAA AGCAGGCACCATG	TCTAGACGTCTCCTCGAGTATCT CTTCCACGCCATGCG
MARCH5	ACGCGTGGTCTCGGATCCAAAAA AGCAGGCACCATG	TCTAGACGTCTCCTCGAGTGCTT CTTCTTGTTCTGGATAA
TRIM29	ACGCGTGGTCTCGGATCCAAAAA AGCAGGCACCATG	TCTAGACGTCTCCTCGAGTGGGG CTTCGTTGGACCCA
TRIM32	ACGCGTGGTCTCGGATCCAAAAA AGCAGGCACCATG	TCTAGACGTCTCCTCGAGTGGGG TGGAATATCTTCTCAG
TRIM44	ACGCGTGGTCTCGGATCCAAAAA AGCAGGCACCATG	TCTAGACGTCTCCTCGAGTGTGT CCTCTTCTTCACTGG
OTUB1	ACGCGTGGTCTCGGATCCAAAAA AGCAGGCACCATG	TCTAGACGTCTCCTCGAGTTTGT AGAGGATATCGTAGTGT

Supplementary Table 4. Human and mouse primers for qPCR.

Gene	Forward (5'-3')	Reverse (5'-3')
RNF13	GAACCCATAGTGCCTCCACC	TCTTTAGTACCTCAATGTCGTTG G
ACTB	CATGTACGTTGCTATCCAGGC	CTCCTTAATGTCACGCACGAT
Acaca	GGCCAGTGCTATGCTGAGAT	AGGGTCAAGTGCTGCTCCA
Srebf1	GACCCTACGAAGTGCACACA	TCTGAGGGTGGAGGGGTAAG
Scd1	TCTTCCTTATCATTGCCAACAC CA	GCGTTGAGCACCAGAGTGTATC G
Fasn	TGGGTTCTAGCCAGCAGAGT	ACCACCAGAGACCGTTATGC
Pparg	ATTCTGGCCCACCAACTTCGG	TGGAAGCCTGATGCTTTATCCCC A
Cd36	GACTGGGACCATTGGTGATGA	AAGGCCATCTCTACCATGCC
Cpt1a	AGGACCCTGAGGCATCTATT	ATGACCTCCTGGCATTCTCC
Ppara	TATTCGGCTGAAGCTGGTGTAC	CTGGCATTTGTTCCGGTTCT
Tnfa	CATCTTCTCAAAATTCGAGTGA CAA	TGGGAGTAGACAAGGTACAACC C
Cxcl10	ATGACGGGCCAGTGAGAATG	ATGATCTCAACACGTGGGCA
Cxcl2	GCGCCCAGACAGAAGTCATA	CAGTTAGCCTTGCCTTTGTTCA
Ccl2	TACAAGAGGATCACCAGCAGC	ACCTTAGGGCAGATGCAGTT
Ccl5	TGCTGCTTTGCCTACCTCTC	TCTTCTCTGGGTTGGCACAC
Col1a1	TGCTAACGTGGTTCGTGACCGT	ACATCTTGAGGTCGCGGCATGT
Col3a1	ACGTAAGCACTGGTGGACAG	CCGGCTGGAAAGAAGTCTGA

Acta2	CCCAGACATCAGGGAGTAATGG	TCTATCGGATACTTCAGCGTCA
Ctgf	TGACCCCTGCGACCCACA	TACACCGACCCACCGAAGACACAG
Timp1	GAGACCACCTTATACCAGCGTT	TACGCCAGGGAACCAAGAAG
Il6	TAGTCCTTCCTACCCCAATTTCC	TTGGTCCTTAGCCACTCCTTC
Il1β	CCGTGGACCTTCCAGGATGA	GGGAACGTCACACACCAGCA
Cxcl12	TGACGGTAAACCAGTCAGCC	CGTGCAACAATCTGAAGGGC
Actb	GTGACGTTGACATCCGTAAAGA	GCCGGACTCATCGTACTCC
Ifnβ	GGATCCTCCACGCTGCGTTCC	CCGCCCTGTAGGTGAGGTTGA

Supplementary Table 5. Antibodies used in the research.

Antibody (dilution/amount)	Source	Identifier
Anti-RNF13 (WB, 1: 500)	Abclonal	A8363
Anti-RNF13 (IHC, 1: 100; WB, 1: 500)	Abcam	ab151601
Anti-LAMP1 (IF, 1: 100)	Cell Signaling Technology	9091
Anti-CD11b (IF, 1: 100)	Boster	BM3925
Anti-CD11b (IHC, 1: 100)	Servicebio	GB11058
Goat anti-mouse IgG-HRP (WB, 1: 10000)	Jackson	115-035-003
Goat anti-rabbit IgG-HRP (WB, 1: 10000)	Jackson	111-035-003
Alexa Flour 568 goat anti-Rabbit IgG (H+L) (IF, 1: 200)	Invitrogen	A11036
Alexa Flour 488 goat anti-Rabbit IgG (H+L) (IF, 1: 200)	Invitrogen	A11034
Alexa Flour 488 goat anti-mouse IgG (H+L) (IF, 1: 200)	Invitrogen	A11029
Alexa Flour 568 donkey anti- goat IgG (H+L) (IF, 1: 200)	Invitrogen	A11057
Anti-STING (WB, 1: 1000)	Cell Signaling Technology	13647
Anti-TBK1 (WB, 1: 500)	Abclonal	A3458
Anti-p-TBK1 (WB, 1: 1000)	Cell Signaling Technology	5483

Anti-IκBα (WB, 1: 1000)	Cell Signaling Technology	4814
Anti-p65 (WB, 1: 500)	Abclonal	A19653
Anti-p-p65 (WB, 1: 1000)	Cell Signaling Technology	3033
Anti-HA (Rabbit mAb) (WB, 1: 1000)	Cell Signaling Technology	3724
Anti-HA (Mouse mAb) (IP, 2 μg/sample)	Medical & Biological Laboratories	M180-3
Anti-Flag (Mouse mAb) (IP, 2 μg/sample)	Medical & Biological Laboratories	M185
Anti-Flag (Rabbit pAb) (WB, 1: 1000)	Cell Signaling Technology	14793
Anti-Myc (Mouse mAb) (WB, 1: 1000)	Medical & Biological Laboratories	M047-3
Anti-β-Actin (WB, 1: 1000)	Abclonal	AC026
Anti-GAPDH (WB, 1: 1000)	Cell Signaling Technology	5174
