# Supplementary material for: RING finger protein 13 protects against nonalcoholic steatohepatitis by targeting STING-relayed signaling pathways
Source: Nat Commun. 2023 Oct 20;14:6635. doi: 10.1038/s41467-023-42420-1 (PMC10587083; doi:10.1038/s41467-023-42420-1)
Supplement: Supplementary file 3 — Reporting Summary [file 41467_2023_42420_MOESM3_ESM.pdf]

nature portfolio

Corresponding author(s): Lin Wang
Last updated by author(s): 9/16/2023

Reporting Summary

Nature Portfolio wishes to improve the reproducibility of the work that we publish. This form provides structure for consistency and transparency in reporting. For further information on Nature Portfolio policies, see our Editorial Policies and the Editorial Policy Checklist.

Statistics

For all statistical analyses, confirm that the following items are present in the figure legend, table legend, main text, or Methods section.

n/a	Confirmed	
☐	☒	The exact sample size (*n*) for each experimental group/condition, given as a discrete number and unit of measurement
☐	☒	A statement on whether measurements were taken from distinct samples or whether the same sample was measured repeatedly
☐	☒	The statistical test(s) used AND whether they are one- or two-sided<br>*Only common tests should be described solely by name; describe more complex techniques in the Methods section.*
☒	☐	A description of all covariates tested
☐	☒	A description of any assumptions or corrections, such as tests of normality and adjustment for multiple comparisons
☐	☒	A full description of the statistical parameters including central tendency (e.g. means) or other basic estimates (e.g. regression coefficient) AND variation (e.g. standard deviation) or associated estimates of uncertainty (e.g. confidence intervals)
☐	☒	For null hypothesis testing, the test statistic (e.g. *F*, *t*, *r*) with confidence intervals, effect sizes, degrees of freedom and *P* value noted<br>*Give P values as exact values whenever suitable.*
☒	☐	For Bayesian analysis, information on the choice of priors and Markov chain Monte Carlo settings
☒	☐	For hierarchical and complex designs, identification of the appropriate level for tests and full reporting of outcomes
☒	☐	Estimates of effect sizes (e.g. Cohen's *d*, Pearson's *r*), indicating how they were calculated

Our web collection on statistics for biologists contains articles on many of the points above.

Software and code

Policy information about availability of computer code

Data collection	Biochemical analysis: ADVIA 2400 Chemistry System Analyzer (Siemens, Tarrytown, NY, USA)<br>Histological and immunohistochemical images: Light microscope (ECLIPSE 80i; Nikon; Tokyo, Japan)<br>Immunofluorescence staining: Confocal laser scanning microscope (TCS SP8; Leica; Wetzler, Germany); High-content analysis system (PerkinElmer; Waltham, MA, USA)<br>qPCR: Real-Time PCR System (LightCycler 480 Instrument II, Roche; Basel, BS, Switzerland)<br>RNA-seq: MGISEQ 2000 (MGI Tech; Shenzhen, China); HISAT2 software (version 2.1.0); SAMtools software (version 1.4); StringTie software (version 1.3.3b); DESeq2 software (version 1.2.10); Java GSEA (version 3.0) platform<br>Western blot: ChemiDoc MP Imaging System (Bio-Rad; Hercules, CA, USA)
Data analysis	Statistical analyses: SPSS 23.0.; GraphPad Prism 8.0<br>Western blot: Image Lab 6.1.0<br>Immunofluorescence images: ImageJ-Fiji 2.9.0

For manuscripts utilizing custom algorithms or software that are central to the research but not yet described in published literature, software must be made available to editors and reviewers. We strongly encourage code deposition in a community repository (e.g. GitHub). See the Nature Portfolio guidelines for submitting code & software for further information.

Data

Policy information about availability of data

All manuscripts must include a data availability statement. This statement should provide the following information, where applicable:

- Accession codes, unique identifiers, or web links for publicly available datasets
- A description of any restrictions on data availability
- For clinical datasets or third party data, please ensure that the statement adheres to our policy

All data are available in the main text or the supplementary materials. Source data are provided with this paper. The RNA-Seq data generated in this study have been deposited in the National Center for Biotechnology Information BioProject database under accession code PRJNA1020209.

Human research participants

Policy information about studies involving human research participants and Sex and Gender in Research.

Reporting on sex and gender	The sex of human participants has been described in Table S1.
Population characteristics	The population characteristics of human participants has been described in Table S1.
Recruitment	Human liver samples were acquired from individuals underwent hepatic surgery at the Department of Hepatobiliary Surgery, Xijing Hospital of the Fourth Military Medical University, and Zhongnan Hospital of Wuhan University in recent years. However, unavoidable operational errors during sample collecting may make results inconsistent with the actual degree of disease in patients, which could make the results slightly biased from the actual situation.
Ethics oversight	Application of human samples were approved and supervised by the ethics committee of Xijing Hospital of Fourth Military Medical University and Zhongnan Hospital of Wuhan University.

Note that full information on the approval of the study protocol must also be provided in the manuscript.

Field-specific reporting

Please select the one below that is the best fit for your research. If you are not sure, read the appropriate sections before making your selection.

[x] Life sciences [ ] Behavioural & social sciences [ ] Ecological, evolutionary & environmental sciences

For a reference copy of the document with all sections, see nature.com/documents/nr-reporting-summary-flat.pdf

Life sciences study design

All studies must disclose on these points even when the disclosure is negative.

Sample size	No sample-size calculation was performed. Sample size was estimated on the basis of previous studies (doi: 10.1016/j.cmet.2020.03.007; 10.1016/j.cmet.2021.05.019) using similar methods and analyses.
Data exclusions	No data were excluded from the analyses in this study.
Replication	Details of biological replicates are provided in figure legends.
Randomization	Allocation was random.
Blinding	All of the animal experiments and a part of in vitro experiments were carried out in a blinded fashion. And all of the in vitro experiments were independently performed by 2-3 individuals, for at least 3 times.

Reporting for specific materials, systems and methods

We require information from authors about some types of materials, experimental systems and methods used in many studies. Here, indicate whether each material, system or method listed is relevant to your study. If you are not sure if a list item applies to your research, read the appropriate section before selecting a response.

Materials & experimental systems

n/a	Involved in the study
☐	☒ Antibodies
☐	☒ Eukaryotic cell lines
☒	☐ Palaeontology and archaeology
☐	☒ Animals and other organisms
☒	☐ Clinical data
☒	☐ Dual use research of concern

Methods

n/a	Involved in the study
☒	☐ ChIP-seq
☒	☐ Flow cytometry
☒	☐ MRI-based neuroimaging

Antibodies

Antibodies used

Antibody (dilution/amount) Source Identifier
Anti-RNF13 (WB, 1: 500) Abclonal A8363
Anti-RNF13 (IHC, 1: 100; WB, 1: 500) Abcam ab151601
Anti-LAMP1 (IF, 1: 100) Cell Signaling Technology 9091
Anti-CD11b (IF, 1: 100) Boster BM3925
Anti-CD11b (IHC, 1: 100) Servicebio GB11058
Goat anti-mouse IgG-HRP (WB, 1: 10000) Jackson 115-035-003
Goat anti-rabbit IgG-HRP (WB, 1: 10000) Jackson 111-035-003
Alexa Flour 568 goat anti-Rabbit IgG (H+L) (IF, 1: 200) Invitrogen A11036
Alexa Flour 488 goat anti-Rabbit IgG (H+L) (IF, 1: 200) Invitrogen A11034
Alexa Flour 488 goat anti-mouse IgG (H+L) (IF, 1: 200) Invitrogen A11029
Alexa Flour 568 donkey anti- goat IgG (H+L) (IF, 1: 200) Invitrogen A11057
Anti-STING (WB, 1: 1000) Cell Signaling Technology 13647
Anti-TBK1 (WB, 1: 500) Abclonal A3458
Anti-p-TBK1 (WB, 1: 1000) Cell Signaling Technology 5483
Anti-IκBα (WB, 1: 1000) Cell Signaling Technology 4814
Anti-p65 (WB, 1: 500) Abclonal A19653
Anti-p-p65 (WB, 1: 1000) Cell Signaling Technology 3033
Anti-HA (Rabbit mAb) (WB, 1: 1000) Cell Signaling Technology 3724
Anti-HA (Mouse mAb) (IP, 2 μg/sample) Medical & Biological Laboratories M180-3
Anti-Flag (Mouse mAb) (IP, 2 μg/sample) Medical & Biological Laboratories M185
Anti-Flag (Rabbit pAb) (WB, 1: 1000) Cell Signaling Technology 14793
Anti-Myc (Mouse mAb) (WB, 1: 1000) Medical & Biological Laboratories M047-3
Anti-β-Actin (WB, 1: 1000) Abclonal AC026
Anti-GAPDH (WB, 1: 1000) Cell Signaling Technology 5174

Validation

The validation statements on the manufacturer website, relevant citations, and antibody profile in online databases can be checked by visiting the company's official website.

Eukaryotic cell lines

Policy information about cell lines and Sex and Gender in Research

Cell line source(s)

Human embryonic kidney 293 (GNHu43) and 293T (GNHu17) cells were purchased from the Cell Bank of the Type Culture Collection of the Chinese Academy of Sciences, Shanghai, China. The human liver cancer cell lines Huh7 (GDC0134) and HepG2 (GDC0024) were purchased from the China Center for Type Culture Collection, Wuhan, China.

Authentication

Cell lines were authenticated by the institutions where they were purchased through STR profiling.

Mycoplasma contamination

All the cell lines were free of mycoplasma contamination.

Commonly misidentified lines
(See ICLAC register)

None.

Animals and other research organisms

Policy information about studies involving animals; ARRIVE guidelines recommended for reporting animal research, and Sex and Gender in Research

Laboratory animals

C57BL/6J male mice aged 6-8 weeks were included in this study, and housed in pathogen-free conditions with a 12-hour light/dark cycle and temperature kept at 22-24°C, humidity kept at 40%-70%. To establish a NAFL model, mice were fed with a high fat diet (HFD; protein, 20%; fat, 60%; carbohydrates, 20%; H10060; HUAFUKANG Bioscience; Beijing, China) for 24 weeks. To establish a NASH model, mice were fed with a high-fat and high-cholesterol diet (HFHC; protein, 14%; fat, 42%; carbohydrates, 44%; cholesterol, 0.2%; TP 26304; Trophic Diet; Nantong, China) for 16 weeks. The mice in the control group were fed with a normal chow diet (NCD;

protein, 18.3%; fat, 10.2%; carbohydrates, 71.5%; 1010001; XIETONG BIO-ENGINEERING; Jiangsu, China) for corresponding time durations.
Rnf13Flox/Flox mice were generated using a CRISPR/Cas9 system in the C57BL/6 background. Rnf13Flox/Flox mice were crossed to albumin-Cre transgenic mice (003574; Jackson Laboratory; Bar Harbor, ME, USA) to generate hepatocyte-specific Rnf13-knockout (Rnf13HKO) mice. For the generation of Rnf13HepTg mice, a Sleeping Beauty (SB) transposase system was applied. To overexpress RNF13 and STING in hepatocytes, we injected two kinds of adeno-associated virus, namely AAV8-TBG-ZsGreen-Rnf13 and the AAV8-TBG-mCherry-Sting1, which were constructed by Hanbio Tech (Shanghai, China), into mice via tail vein before HFHC feeding and after 8-week of HFHC feeding.

Wild animals	None.
Reporting on sex	Only male mice were used in the study.
Field-collected samples	None.
Ethics oversight	Animal experiments were approved by the Animal Care and Use Committee of Fourth Military Medical University.

Note that full information on the approval of the study protocol must also be provided in the manuscript.
